# Impaired sensitivity to thyroid hormones is associated with high lipoprotein(a) level in euthyroid patients with type 2 diabetes mellitus

**DOI:** 10.3389/fendo.2025.1591108

**Published:** 2025-06-25

**Authors:** Luojing Zhong, Ruiyu Lin, Baozhen Cao, Wenying Zhong, Mei Tu, Wen Wei

**Affiliations:** ^1^ Department of Endocrinology, Longyan First Affiliated Hospital of Fujian Medical University, Longyan, China; ^2^ Department of Physical Examination, Longyan First Affiliated Hospital of Fujian Medical University, Longyan, China; ^3^ The School of Clinical Medicine, Fujian Medical University, Fuzhou, China

**Keywords:** lipoprotein(a), sensitivity to thyroid hormones, type 2 diabetes mellitus, thyroid feedback quantile-based index, free triiodothyronine/free thyroxine ratio

## Abstract

**Background:**

The relationship between thyroid hormone sensitivity and lipoprotein(a) (Lp(a)) is unclear. The purpose of this study is to illuminate the correlation between thyroid hormone sensitivity and Lp(a) in euthyroid patients with type 2 diabetes mellitus (T2DM).

**Method:**

A cross-sectional study was conducted on 1097 patients with T2DM. The thyroid hormone sensitivity indices, the thyroid feedback quantile-based index (TFQI), the thyroid-stimulating hormone index (TSHI), the thyrotrophic T4 resistance index (TT4RI), and the free triiodothyronine(FT3)/free thyroxine (FT4) ratio were calculated. Linear and binary logistic regression analysis were performed to assess the relationship between thyroid hormone sensitivity indices and Lp(a). Restricted cubic splines were also used to evaluate the association between thyroid hormone sensitivity indices and Lp(a).

**Result:**

Among the 1097 participants, the percentage of Lp(a)≥30 mg/dL was 20.3%. Linear regression analysis revealed that TFQI displayed a positive correlation with Lp(a) levels, whereas the FT3/FT4 ratio exhibited a negative correlation. The adjusted odds ratio (aOR)(95% confidence interval(CI)) for high Lp(a) level was increased with rising TFQI quartiles(Q3: aOR 1.49,95% CI 1.09-2.06; Q4: aOR 1.74, 95% CI 1.36-2.23) (*P*<0.05),with Q1 as the reference. By contrast,the aOR(95% CI) for high Lp(a) level was decreased with rising FT3/FT4 ratio quartiles (*P*<0.05). The robustness of these findings was further confirmed by restricted cubic spline analysis.

**Conclusion:**

In euthyroid T2DM patients, decreased sensitivity to thyroid hormones was found to be linked with high Lp(a) level. Screening for thyroid hormone insensitivity and serum Lp(a) levels should be emphasized in euthyroid T2DM patients for early intervention and improved outcomes.

## 1 Introduction

Thyroid hormones are crucial determinants of overall energy expenditure and significant regulators of various lipid metabolic processes (1, 2).Numerous studies have demonstrated a causal relationship between thyroid dysfunction and dyslipidemia (1, 3, 4). However, prior research has indicated that thyroid hormone or thyroid-stimulating hormone (TSH) levels alone may not fully explain the relationship between the thyroid system and dyslipidemia (3–5), and comprehensive indices can systematically reflect thyroid hormone homeostasis regulation (6). Indices such as the TSH index (TSHI), thyrotrophic T4 resistance index (TT4RI), and thyroid feedback quantile-based index (TFQI) have been validated for assessing central sensitivity to thyroid hormones. By comprehensively considering the levels of TSH and thyroid hormones, these indices can more comprehensively reflect the feedback regulatory effects of thyroid hormones on the hypothalamus and pituitary gland (7). The free triiodothyronine (FT3)/free thyroxine (FT4) ratio reflects the peripheral bioavailability of thyroid hormones, potentially being a more precise and feasible indicator of thyroid hormone metabolic variability than FT3 or FT4 alone (8). A growing body of studies have shown that higher values of these composite indices are associated with dyslipidemia, metabolic syndrome (MS), nonalcoholic fatty liver disease(NAFLD), carotid plaque, and atherosclerosis (AS), even in euthyroid populations (6, 9–13). Duan et al. reported that the risk of resistance to thyroid hormones was positively correlated with high non-high-density lipoprotein cholesterol (non-HDL-C) levels in patients with type 2 diabetes mellitus (T2DM) (14). Non-HDL-C, calculated as total cholesterol (TC) minus high-density lipoprotein cholesterol (HDL-C), includes all plasma lipoproteins, such as low-density lipoprotein cholesterol (LDL-C), triglyceride (TG)-rich lipoprotein (TRL), TRL-remnants, and lipoprotein(a) (Lp(a)) (15). The findings indicates the pathogenic effect of reduced sensitivity to thyroid hormones on serum lipid metabolism.

Dyslipidemia and T2DM represent chronic conditions with profound public health implications (16). Among individuals with T2DM, the incidence of dyslipidemia is high. A study conducted in Ethiopia revealed a 59% prevalence of dyslipidemia among patients with T2DM (17), whereas research in Kenya observed an even higher rate of 86.1% among diabetic patients (18). Disordered lipid metabolism is a major contributor to atherosclerotic cardiovascular disease(ASCVD) risk in T2DM patients (19), who face approximately double the ASCVD risk compared to non-diabetic patients (20). ASCVD, a vascular complication of T2DM, is a leading cause of mortality. Despite the relatively good control of common risk factors, such as hyperlipidemia, hyperglycemia, hypertension, and smoking, the incidence of cardiovascular events remains high in patients with T2DM. It suggests the presence of residual cardiovascular risk (21). Dyslipidemia commonly observed in T2DM patients is characterized by elevated LDL-C, low HDL-C, and elevated TG. In addition to these common dyslipidemias, the fourth “clinical” category of lipid disorders, elevated Lp(a), constitutes an important component of residual cardiovascular risk in T2DM (22).

Lp(a), an LDL-cholesterol-like particle first identified by Dr. Kåre Berg in 1963 (23). Its concentration is predominantly genetically determined, exhibiting significant variations across populations (24). Though no universally recognized absolute risk threshold exists, approximately 20% to 25% of the global population has Lp(a) levels of 50 mg/dL or higher (24), conferring an elevated cardiovascular risk despite optimizing traditional risk factors (25), according to the European Atherosclerosis Society (EAS). High Lp(a) level constitute an independent risk factor for cardiovascular disease, capable of increasing the risk even when LDL-C levels are within the recommended range (21). Hence, screening patients with elevated Lp(a) can identify those needing more intensive cardiovascular risk management.This study aims to illuminate the correlation between thyroid hormone sensitivity and Lp(a) in euthyroid T2DM patients, providing new evidence for the role of impaired thyroid hormone sensitivity for serum atherogenic Lp(a) levels.

## 2 Methods

### 2.1 Study population

**Figure 1:**
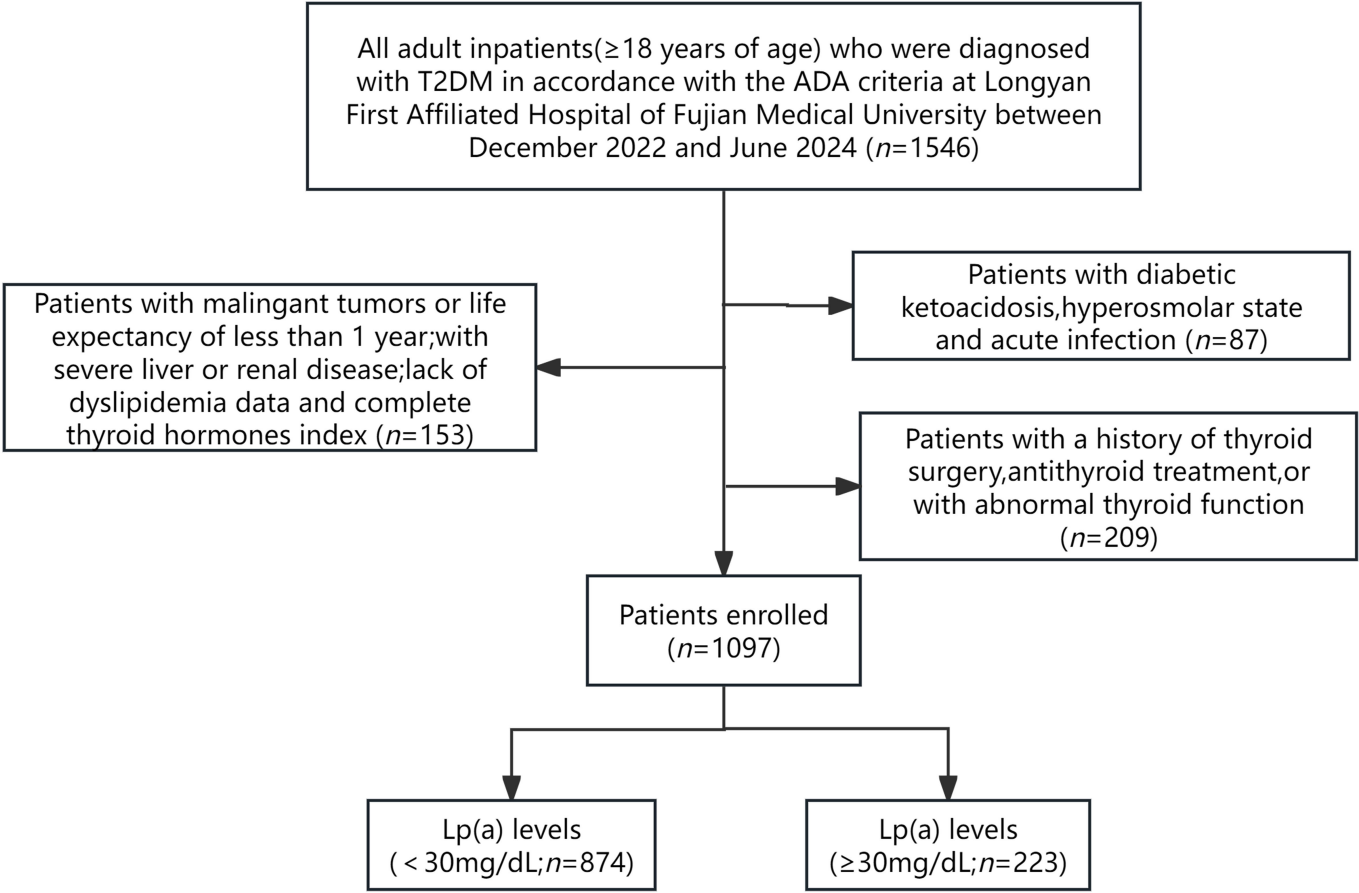
The flow of participants through the trial.

The current investigation was conducted as a cross-sectional study that included adult inpatients(≥18 years of age) who were diagnosed with T2DM in accordance with the criteria outlined by the American Diabetes Association (ADA) at Longyan First Affiliated Hospital of Fujian Medical University,Fujian,China between December 2022 and June 2024. The exclusion criteria included: (1) pregnant patients and those with other diabetes types; (2) individuals with acute complications of diabetes, such as diabetic ketoacidosis, hyperosmolar state and acute infection; (3) patients with maglingant tumors or life expectancy of less than 1 year; (4) patients with severe liver or renal diseases; (5) lack of essential data on FT3, FT4, TSH and Lp(a); (6) a history of thyroid surgery, antithyroid treatment, or with abnormal thyroid function. Ultimately, 1097 patients were recruited for the study (**Figure 1**).

### 2.2 Clinical and biochemical measurements

Clinical data and biochemical examination results were received from the electronic health record system.Upon admission,standardized measurements of height,weight, and blood pressure (BP) were conducted by the nurse. After an overnight fast, blood samples were collected from participants and analyzed in the biochemistry laboratory at Longyan First Affiliated Hospital of Fujian Medical University. The biochemical parameters evaluated included fasting blood glucose (FBG),glycosylated hemoglobin (HbA1c), TC, TG, HDL-C, LDL-C, Lp(a), alanine aminotransferase(ALT), aspartate aminotransferase(AST),and serum creatinine.

Body mass index (BMI) was computed by dividing weight (kg) by the square of height (m). The estimated glomerular filtration rate (eGFR) was calculated using the serum creatinine level and the Chronic Kidney Disease Epidemiology Collaboration (CKD-EPI) 2009 equation (26). The diagnosis of diabetic retinopathy (DR) was established based on ophthalmological examination and adherence to the International Clinical Diabetic Retinopathy Disease Severity Scale (27). Diabetic nephropathy (DN) was diagnosed when the urinary albumin/creatinine ratio(ACR) was ≥30 mg/mmol or eGFR was <60 mL/min/1.73m², as defined by Kidney Disease improving Global Outcomes (KDIGO) (26, 28). Diabetic peripheral neuropathy (DPN) was diagnosed according to the Chinese guidelines for managing type 2 diabetes mellitus (29). Cardiovascular disease (CVD) encompassed stroke and/or coronary artery disease (CAD).

### 2.3 Measurement of Lp(a)

Upon admission, lipid profiles were assessed, with Lp(a) levels measured using an auto-immunoturbidimetry assay on an AU5800 Analyzer (Beckman Coulter, Brea, California). The intra-assay and inter-assay coefficients of variation were ≤4% and ≤10%, respectively. Based on previous literature (30) and the manufacturer’s instructions for our hospital’s Lp(a) kit, Lp(a) levels <30 mg/dL were deemed normal. We defined the Lp(a) levels ≥30 mg/dL as high Lp(a) level in this study.

### 2.4 Measurements of thyroid parameters

TSH levels were assayed using a third-generation immunoassay, while FT3 and FT4 levels were determined via competitive immunoassay methods. The reference intervals for FT3, FT4, and TSH were 3.53-7.37 pmol/L,7.98-16.02 pmol/L, and 0.56-5.91 mIU/L,respectively. Central indices of thyroid hormone sensitivity were calculated using the following formulas:

TFQI=cumulative distribution function(CDF)FT4-(1- CDF TSH);

TSHI=Ln TSH (μIU/mL)+0.1345×FT4 (pmol/L);

TT4RI=FT4 (pmol/L)×TSH (μIU/mL).

For TSHI, TT4RI, and TFQI, higher values implied lower central sensitivity to thyroid hormones (6). Conversely, the peripheral index of thyroid hormone sensitivity was calculated as below:

FT3/FT4 ratio= FT3(pmol/L)/FT4(pmol/L).

Higher ratios indicated greater peripheral sensitivity to thyroid hormones.

### 2.5 Statistical analysis

Data were presented as mean ± standard deviation or median (interquartile range) depending on the distribution of the continuous variable, and categorical variables were presented as number(%). The unpaired Student’s t test and the Mann-Whitney U test were utilized to assess differences in normally distributed and skewed distributed variables between the two groups, respectively. The Chi-square test was used for categorical variables, as appropriate.

The analysis of Cochran-Armitage was applied for comparing the levels of Lp(a) and the prevalence of T2DM patients with high Lp(a) level across the quartiles of thyroid parameters (TFQI:-0.861≤Q1<-0.309, -0.309≤Q2<-0.0368, -0.0368≤Q3<0.193, 0.193≤Q4 ≤ 0.961; FT3/FT4 ratio: 0.225≤Q1<0.373, 0.373≤Q2< 0.426,0.426≤Q3<0.483,0.483≤Q4 ≤ 0.676). Restricted cubic splines were used to detect the association between high Lp(a) level and thyroid hormone sensitivity indices. The association between Lp(a) and thyroid-associated variables was assessed by univariable and multivariable linear regression. To further evaluate the potential associations of high Lp(a) level with impaired thyroid hormone sensitivity,univariable and multivariable binary logistic regression model were performed. The results of the logistic regression analysis were presented as ORs and 95%CIs. The variance inflation factor was used to measure whether there was multicollinearity among thyroid-associated variables in the regression model. Model 1:crude model; Model 2:adjusted for age, sex and BMI; Model 3:adjusted for age, sex, BMI, DR, AST, eGFR, TG and HDL-C.

Statistical analyses were performed using R,version 4.0.3 software(R Foundation for Statistical Computing, Vienna, Austria),SPSS version 26.0 (IBM corp,Armonk,NY) and the Graphpad prism 10.1.2 software. Two-sided p values<0.05 were considered statistically significant.

## 3 Results

### 3.1 Baseline characteristics of the participants

A total of 1097 adults included in this study were grouped into two groups (Lp(a)<30 and ≥30mg/dL). The characteristics of the participants according to different Lp(a) levels are shown in **Table 1**. The majority were male (63.0%), and the average age was 57.6 years. The median duration of diabetes was 6.0 years, and the glycosylated hemoglobin (HbA1c) was 9.7% ± 2.3. Almost half of the patients had hypertension (45.9%; *n*=504),26.5% (*n*=273) had DR,13.0% (*n*=143) had DN,35.9% (*n*=394) had DNP, and 11.2% (*n*=123) had CVD.

**Table 1:** Baseline characteristics of the participants.

Characteristic	All (*n*=1097)	Lp(a) levels (mg/dL)		*P* -value
		<30 (*n*=874)	≥30 (*n*=223)	
Demographic characteristics				
Age (years)	57.6 ± 12.1	57.6 ± 12.2	57.7 ± 11.8	0.904
Male,*n* (%)	691 (63.0)	549 (62.8)	142 (63.7)	0.873
BMI (kg/m^2^)	24.3 ± 3.3	24.4 ± 3.3	23.9 ± 3.4	0.046
Medical history and clinical condition				
Hypertension,*n* (%)	504 (45.9)	402 (46.0)	102 (45.7)	1.000
SBP (mmHg)	133.3 ± 18.7	133.2 ± 18.5	133.3 ± 19.5	0.986
DBP (mmHg)	83.0 ± 11.3	83.2 ± 11.5	82.3 ± 10.8	0.281
Duration of diabetes(years)	6.0 (1.0,10.0)	6.0 (0.5,10.0)	7.0 (2.0,10.0)	0.196
DR,*n* (%)	273 (26.5)	212 (25.7)	61 (29.8)	0.280
DN,*n* (%)	143 (13.0)	108 (12.4)	35 (15.7)	0.229
DPN,*n* (%)	394 (35.9)	313 (35.9)	81 (36.3)	0.958
CVD,*n* (%)	123 (11.2)	97 (11.1)	26 (11.7)	0.906
Laboratory examination				
FBG (mmol/L)	8.63 ± 3.20	8.68 ± 3.22	8.45 ± 3.15	0.357
HbA1C (%)	9.70 ± 2.32	9.65 ± 2.30	9.93 ± 2.37	0.114
ALT (U/L)	21.0 (15.0,31.0)	21.0 (15.0,32.0)	19.5 (15.0,27.8)	0.049
AST (U/L)	20.0 (17.0,26.0)	20.0 (17.0,26.0)	20.0 (16.0,25.0)	0.200
Creatinine (umol/L)	69.5 (58.0,85.0)	69.0 (57.5,84.0)	72.0 (59.0,87.0)	0.055
eGFR (mL/min/1.73m^2^)	90.57 ± 23.34	91.32 ± 22.90	87.59 ± 24.79	0.034
TC (mmol/L)	5.23 ± 1.46	5.18 ± 1.45	5.40 ± 1.48	0.044
TG (mmol/L)	1.65 (1.11,2.63)	1.58 (1.11,2.32)	1.68 (1.12,2.76)	0.048
LDL-C (mmol/L)	3.27 ± 0.98	3.23 ± 0.97	3.43 ± 1.02	0.007
HDL-C (mmol/L)	1.11 ± 0.29	1.15 ± 0.28	1.10 ± 0.29	0.031
Medication				
Statin,*n* (%)	122 (12.2)	91 (11.4)	31 (15.5)	0.142
Fibrate,*n* (%)	6 (0.6)	4 (0.5)	2 (1.0)	0.760
Aspirin/Clopidogrel,*n* (%)	94 (9.4)	69 (8.6)	25 (12.5)	0.124
Thyroid function and indices of thyroid hormone sensitivity				
FT3 (pmol/L)	4.95 ± 0.64	4.96 ± 0.64	4.91 ± 0.61	0.240
FT4 (pmol/L)	11.74 ± 1.71	11.66 ± 1.70	12.03 ± 1.72	0.004
TSH (uIU/mL)	1.65 (1.16,2.43)	1.65 (1.16,2.46)	1.61 (1.17,2.27)	0.406
FT3/FT4	0.43 ± 0.08	0.43 ± 0.08	0.42 ± 0.08	0.002
TSHI	2.08 (1.74,2.47)	2.08 (1.73,2.45)	2.08 (1.74,2.51)	0.744
TFQI	-0.04 ± 0.37	-0.06 ± 0.37	-0.01 ± 0.37	0.086

Lp(a), lipoprotein(a); BMI, body mass index; SBP, systolic blood pressure; DBP, diastolic blood pressure; DR, diabetic retinopathy; DN, diabetic nephropathy; DPN, diabetic peripheral neuropathy; CVD, cardiovascular disease; FBG, fasting blood glucose; HbA1c, glycosylated hemoglobin; AST, aspartate aminotransferase; ALT, alanine aminotransferase; eGFR, estimated glomerular filtration rate; TG, triglyceride; TC, total cholesterol; LDL-C, low-density lipoprotein cholesterol; HDL-C, high-density lipoprotein cholesterol; FT3, Free Triiodothyronine; FT4, Free Thyroxine; TSH, Thyroid-stimulating hormone; TSHI, Thyroid-stimulating hormone index; TT4RI, Thyrotrophic T4 resistance index; TFQI, Thyroid feedback quantile-based index.

The percentage of Lp(a)≥30 mg/dL was 20.3% (*n*=223). The levels of BMI, ALT, eGFR and HDL-C were significantly decreased in the high Lp(a) level group and increased in the normal Lp(a) level group (Lp(a)<30mg/dL)(*P*<0.05). Participants with high Lp(a) level exhibited higher TC,TG and LDL-C levels(*P*<0.05). Compared with patients in the Lp(a)<30 mg/dL group, those in the Lp(a)≥30 mg/dL group had higher FT4 levels and lower FT3/FT4 levels(*P*<0.01). The levels of FT3,TSH,TT4RI,TSHI,and TFQI did not differ between the two groups.

**Figure 2:**
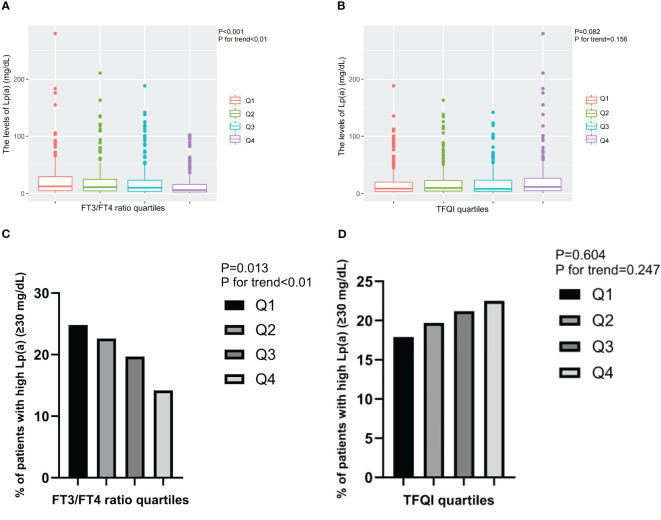
The levels of Lp(a) and the prevalence of T2DM patients with high Lp(a) level across the quartiles of thyroid parameters. **(A)** The levels of Lp(a) across the FT3/FT4 ratio quartiles. **(B)** The levels of Lp(a) across the TFQI quartiles. **(C)** The prevalence of T2DM patients with high Lp(a) level across the FT3/FT4 ratio quartiles. **(D)** The prevalence of T2DM patients with high Lp(a) level across the TFQI quartiles. Q1, the 1^st^ quartiles; Q2, the 2^nd^ quartiles; Q3, the 3^rd^ quartiles; Q4, the 4^th^ quartiles; Lp(a), lipoprotein(a); NS, P>0.05; FT3, Free Triiodothyronine; FT4, Free Thyroxine; TFQI, Thyroid feedback quantile-based index; T2DM, type 2 diabetes mellitus.

Furthermore, the levels of Lp(a) and the prevalence of high Lp(a) level significantly decreased from the lowest to highest quartiles of FT3/FT4 ratio (*P* for trend<0.01), while exhibited increasing trend from the lowest to highest quartiles of TFQI (**Figure 2**).

### 3.2 Association of thyroid related indicators with Lp(a) levels by linear regression analysis

Linear regression analysis indicated that FT4 and TFQI levels were positively associated with Lp(a) levels (**Table 2**) (all *P*<0.05). Age, gender, BMI, TG, HDL-C, DR, AST and eGFR were included as covariates in the multivariable analysis. The positive correlations remained after adjusting for covariates. Whereas, FT3/FT4 ratio levels were negatively associated with Lp(a) levels (all *P*<0.05). The negative relationships remained after full adjustment(all *P*<0.05). The univariable linear regression model between thyroid parameters and Lp(a) levels in euthyroid patients with T2DM was presented in Additional file: **Supplementary Table S1**.

**Table 2:** Linear regression analysis for the association between Lp(a) levels and thyroid parameters in euthyroid patients with type 2 diabetes mellitus.

Variables	Model 1		Model 2		Model 3	
	β (95% CI) *P*-value		β (95% CI) *P*-value		β (95% CI) *P*-value	
FT3	-1.91 (-4.50,0.67)	0.147	-1.15 (-3.91,1.61)	0.413	0.35 (-2.61,3.31)	0.817
FT4	1.21 (0.26,2.17)	0.013	1.18 (0.22,2.15)	0.016	1.37 (0.36,2.38)	0.008
TSH	-0.38 (-1.94,1.18)	0.634	-0.51 (-2.08,1.07)	0.526	-0.18 (-1.81,1.46)	0.832
TFQI	4.26 (0.10,8.24)	0.049	4.72 (0.12,9.32)	0.043	4.87 (0.28,9.46)	0.038
TSHI	1.87 (-1.25,4.98)	0.240	1.68 (-1.46,4.82)	0.294	2.53 (-0.68,5.74)	0.123
TT4RI	0.03 (-0.11,0.17)	0.689	0.02 (-0.12,0.16)	0.789	0.06 (-0.09,0.20)	0.451
FT3/FT4	-31.12 (-51.69,-10.55)	0.003	-26.77 (-47.91,-5.63)	0.013	-23.93 (-46.43,-1.43)	0.037

Model 1:crude model. Model 2:adjusted for age, sex and BMI. Model 3:adjusted for age, sex, BMI, DR, AST, eGFR, TG and HDL-C.

Lp(a), lipoprotein(a); BMI, body mass index; DR, diabetic retinopathy; AST, aspartate aminotransferase; eGFR, estimated glomerular filtration rate; TG, triglyceride; HDL-C, high-density lipoprotein cholestrol; FT3, Free Triiodothyronine; FT4, Free Thyroxine; TSH, Thyroid-stimulating hormone; TSHI, Thyroid-stimulating hormone index; TT4RI, Thyrotrophic T4 resistance index; TFQI, Thyroid feedback quantile-based index.

### 3.3 Association of thyroid related indicators with high Lp(a) level by logistic regression analysis

To explore the association between high Lp(a) level and thyroid related indicators, binary logistic regression analysis was applied for calculation (**Table 3**). For 1 SD increase in FT4 and TFQI,the ORs for high Lp(a) level were 1.16(95% CI 1.05-1.27) and 1.54(95% CI 1.01-2.35) after adjusting for confounding factors, respectively (**Table 3**) (all *P*<0.05). Whereas 1 SD increase in FT3/FT4 ratio had an OR of 0.08(95% CI 0.01-0.66) for high Lp(a) level by full adjustment (**Table 3**) (*P*<0.05). The association of high Lp(a) level with thyroid parameters in euthyroid patients with T2DM of univariable logistic regression model was presented in Additional file: **Supplementary Table S2**.

**Table 3:** Logistic regression analysis for the association between high Lp(a) level and thyroid parameters in euthyroid patients with type 2 diabetes mellitus.

Variables	Model 1		Model 2		Model 3	
	OR (95% CI) *P*-value		OR (95% CI) *P*-value		OR (95% CI) *P*-value	
FT3	0.87 (0.69,1.10)	0.240	0.90 (0.70,1.15)	0.396	1.05 (0.80,1.38)	0.735
FT4	1.13 (1.04,1.23)	0.004	1.12 (1.03,1.22)	0.009	1.16 (1.05,1.27)	0.003
TSH	0.92 (0.79,1.06)	0.244	0.93 (0.80,1.07)	0.331	0.94 (0.80,1.10)	0.454
TFQI	1.51 (1.01,2.30)	0.047	1.52 (1.01,2.33)	0.041	1.54 (1.01,2.35)	0.037
TSHI	1.05 (0.79,1.39)	0.734	1.07 (0.81,1.42)	0.631	1.16 (0.86,1.56)	0.330
TT4RI	1.00 (0.99,1.01)	0.768	1.00 (0.99,1.01)	0.901	1.00 (0.99,1.01)	0.834
FT3/FT4	0.05 (0.01,0.35)	0.003	0.07 (0.01,0.49)	0.008	0.08 (0.01,0.66)	0.021

Model 1:crude model. Model 2:adjusted for age, sex and BMI. Model 3:adjusted for age, sex, BMI, DR, AST, eGFR, TG and HDL-C.

Lp(a), lipoprotein(a); BMI, body mass index; DR, diabetic retinopathy; AST, aspartate aminotransferase; eGFR, estimated glomerular filtration rate; TG, triglyceride; HDL-C, high-density lipoprotein cholestrol; FT3, Free Triiodothyronine; FT4, Free Thyroxine; TSH, Thyroid-stimulating hormone; TSHI, Thyroid-stimulating hormone index; TT4RI, Thyrotrophic T4 resistance index; TFQI, Thyroid feedback quantile-based index.

**Figure 3:**
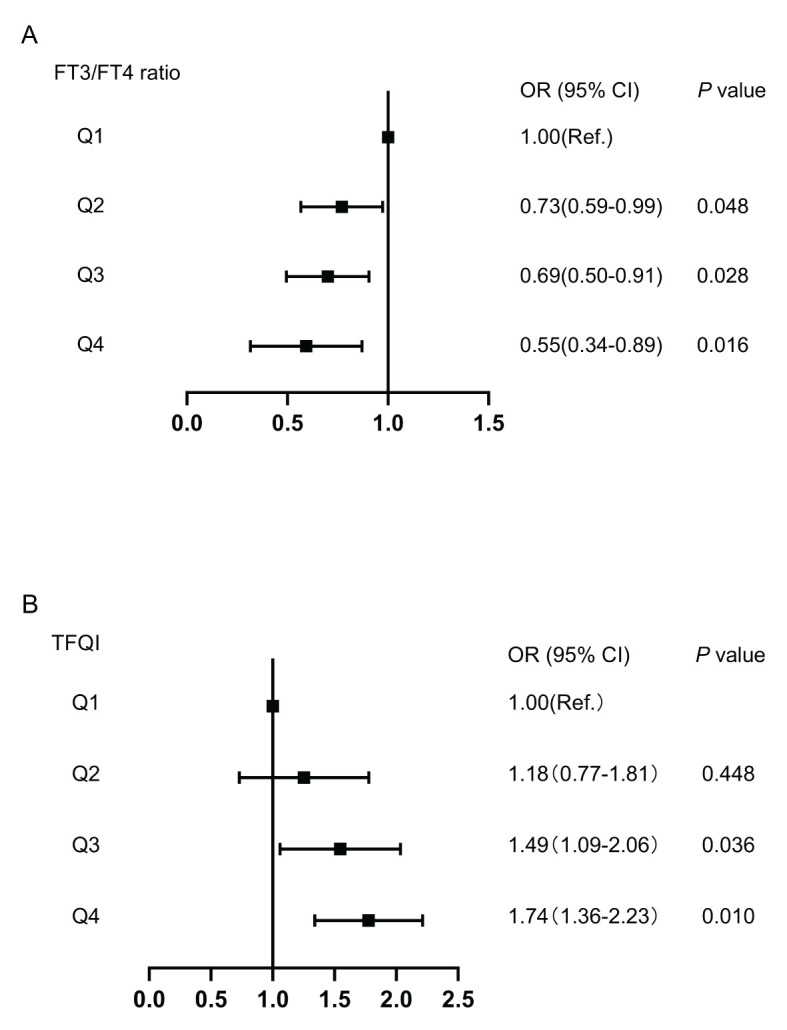
The forest maps of logistic regression analysis for the association between high Lp(a) level and quartiles of FT3/FT4 ratio and TFQI, with the 1^st^ quartile as the reference. **(A)** The ORs for high Lp(a) level across FT3/FT4 ratio quartiles. **(B)** The ORs for high Lp(a) level across TFQI quartiles. Model was adjusted for age, sex, BMI, DR, AST, eGFR, TG and HDL-C. Q1, the 1^st^ quartiles; Q2, the 2^nd^ quartiles;Q3, the 3^rd^ quartiles;Q4, the 4^th^ quartiles; Lp(a), lipoprotein(a); BMI, body mass index; DR, diabetic retinopathy; AST, aspartate aminotransferase; eGFR, estimated glomerular filtration rate; TG, triglyceride; HDL-C, high-density lipoprotein cholesterol; FT3, Free Triiodothyronine; FT4, Free Thyroxine; TFQI, Thyroid feedback quantile-based index.

The associations of TFQI and FT3/FT4 ratio quartiles with high Lp(a) level were shown in **Figure 3**. Compared with the Q1 TFQI levels, the Q3 and Q4 TFQI levels showed positive association with high Lp(a) level after adjustment (Q3: OR 1.49,95% CI 1.09-2.06; Q4: OR 1.74, 95% CI 1.36-2.23) (*P*<0.05). Instead, there was a negative correlation between the FT3/FT4 ratio and high Lp(a) level (Q2: OR 0.73,95% CI 0.59-0.99; Q3: OR 0.69,95% CI 0.50-0.91; Q4: OR 0.55,95% CI 0.34- 0.89)(*P*<0.05). However, no significant associations were observed between high Lp(a) level and FT3,TSH,TSHI and TT4RI.

### 3.4 RCS analysis investigating the relationship between high Lp(a) level and TFQI and FT3/FT4 ratio

**Figure 4:**
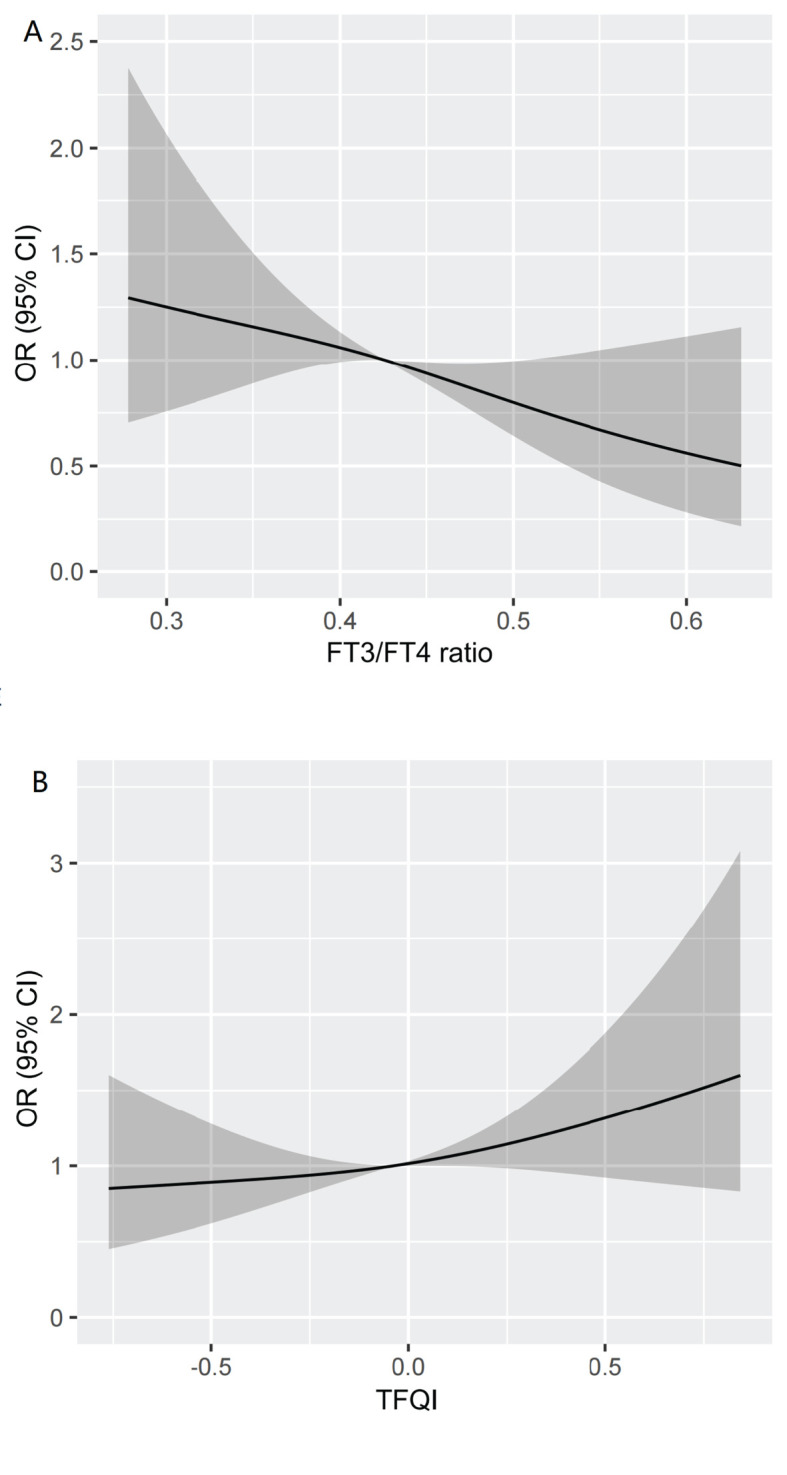
Restricted spline curve of the FT3/FT4 ratio and TFQI odds ratio of high Lp(a) level after adjusting for the covariates including Age, gender, BMI, TG,HDL-C,DR,AST and eGFR. **(A)** The restricted spline curve of the FT3/FT4 ratio odds ratio of high Lp(a) level. **(B)** The restricted spline curve of the TFQI odds ratio of high Lp(a) level. Abbreviations: Lp(a), lipoprotein(a); BMI, body mass index; DR, diabetic retinopathy; AST,aspartate aminotransferase; eGFR, estimated glomerular filtration rate; TG, triglyceride; HDL-C, high-density lipoprotein cholesterol; FT3, Free Triiodothyronine; FT4, Free Thyroxine; TFQI, Thyroid feedback quantile-based index.

We visualized the association between high Lp(a) level and TFQI and FT3/FT4 ratio using RCS analysis in **Figure 4**. After adjusting for the covariates including age, gender, BMI, TG, HDL-C, DR, AST and eGFR, there was a linear correlation between high Lp(a) level and thyroid hormone sensitivity indices in our study population. We found that the higher the FT3/FT4 ratio, the lower the high Lp(a) level. Conversely, the higher the TFQI, the higher the high Lp(a) level (nonlinear: FT3/FT4 ratio, *P*=0.6451;TFQI, *P*=0.6585). The restricted spline curves of the FT3/FT4 ratio and TFQI odds ratio of high Lp(a) level of univariable logistic regression model were presented in Additional file: **Supplementary Figure S1**.

## 4 Discussion

To the best of our knowledge, this present study was the first study to investigate the association between thyroid hormone sensitivity and Lp(a) levels in euthyroid patients with T2DM. We found that Lp(a) levels were significantly positively associated with TFQI. Conversely, a negative association was noted between the Lp(a) levels and FT3/FT4 ratio, even after adjusting for multiple confounding factors, suggesting that increased Lp(a) levels were associated with decreased sensitivity to thyroid hormones in euthyroid patients with T2DM.

The association between thyroid hormone sensitivity and lipid metabolism was investigated in previous studies. Liu et al (31) conducted an analysis of the correlations between thyroid hormone sensitivity indices and lipid parameters (including TC, TG, HDL-C, and LDL-C), revealing that the likelihood of dyslipidemia positively aligns with TFQI, TSHI, and TT4RI, while inversely correlates with the FT3/FT4 ratio in individuals with coronary heart disease. Duan et al (14) have documented that the likelihood of elevated non-HDL-C levels exhibits a direct relationship with TFQI,TSHI, and TT4RI, while simultaneously demonstrating an inverse association with the FT3/FT4 ratio in T2DM patients. A recent study, involving a substantial sample size, further suggested that among euthyroid adults, a diminished sensitivity to thyroid hormones is linked to elevated RC levels (12). Hence, thyroid hormone sensitivity emerges as a significant factor in relation to dyslipidemia, indicating that periodic screening of thyroid hormones sensitivity indices in euthyroid patients with T2DM is recommended to facilitate early intervention of dyslipidemia. In line with previous studies, our study revealed that TFQI displayed a positive correlation with Lp(a) levels, whereas the FT3/FT4 ratio exhibited a negative correlation. We also found that in euthyroid patients with T2DM, the prevalence and risk of high Lp(a) level increased as thyroid hormone sensitivity decreased. For instance, for each one-unit increase in the TFQI, the risk of high Lp(a) level increases by 54%.Conversely, for each one-unit increase in the FT3/FT4 ratio, the risk of high Lp(a) level decreases by 8%. Lp(a) in serum constitutes a unique lipoprotein produced by hepatic synthesis. This lipoprotein is characterized as a heterogeneous glycoprotein, specifically an apoB100-containing lipoprotein that undergoes covalent attachment to apoprotein(a) (32). Numerous studies have provided compelling evidence suggesting that an increased level of Lp(a), serves as a primary contributor to the risk of ASCVD, myocardial infarction, and stenosis of the aortic valve (33). The results of our study provided additional support for thyroid hormone resistance as a standalone risk factor contributing to atherogenic lipid profiles and unfavorable cardiovascular outcomes. Consequently, timely identification of thyroid hormone resistance or lipid abnormalities is crucial in prevention and treatment approaches, and reducing the incidence and advancement of these conditions in euthyroid patients with T2DM.

In our research, we observed elevated TFQI levels in individuals with high Lp(a) level, suggesting a state of central resistance to thyroid hormones. To demonstrate the presence of mild central thyroid hormone resistance, we employed three composite indices in this study. Notably, TFQI, a novel index first introduced by Laclaustra et al. in 2019 for detecting acquired thyroid hormone resistance (6), exhibiting greater stability compared to TSHI and TT4RI in assessing thyroid hormone sensitivity (6). Beyond its strong correlation with diabetes and metabolic syndrome, a prospective study revealed that TFQI was independently associated with all-cause mortality in euthyroid individuals (34). In our study, the level of TSHI and TT4RI did not differ among euthyroid patients with T2DM with or without lipid abnormality. This could be attributed to TFQI as a better induce in assessing central sensitivity to thyroid hormones compared to TSHI and TT4RI,and further research is needed to verify this issue. Previous studies have indicated that relatively high TSH levels was positively associated with unfavorable lipid concentration and cardiovascular risks in enthyroid people (2, 35). This is differ to our findings. The differences in research results may be attributable to differences in study populations, sample sizes, and confounding factors. Therefore, it is recommended that euthyroid patients with T2DM undergo regular screening for central sensitivity to thyroid hormones, especially the level of TFQI, to minimize the potential for unfavorable clinical occurrences.

Clinically, thyroid function is assessed by measuring serum levels of FT3, FT4, and TSH. TSH promotes the synthesis and release of FT4. Subsequently, FT4, which affects TSH production by the pituitary gland through a feedback loop, is converted into the active FT3 hormone in thyroid and peripheral tissues, and the FT3/FT4 ratio reflects the proportion of this conversion (36). As such, the two-way feedback regulation of the hypothalamus-pituitary-thyroid (HPT) axis results in relatively complex effects of thyroid hormones (37). In our study, FT4 levels were higher in the high Lp(a) level group and FT4 levels were an independent risk factor for high levels of Lp(a). In our study, neither TSH nor FT3 levels were significantly correlated with the levels of Lp(a). This indicates a stronger correlation between FT4 and Lp(a) levels, compared to FT3 and TSH. As a marker indicating the conversion rate from FT4 to FT3, FT3/FT4 ratio was observed to negatively related to the Lp(a) levels, which could verify the speculation that FT4 exerts more pathogenic effects in the occurrence and aggravation of lipid abnormality. Hence, the euthyroid patients with T2DM should pay attention to the detection of FT4 levels to reduce the risk of adverse clinical events.

The strength of our research is the presentation of further evidence indicating central and peripheral thyroid hormone resistance as an independent risk factor for elevated Lp(a) concentrations in euthyroid patients with T2DM, which holds significant implications for T2DM patients at heightened risk of ASCVD. It is essential to acknowledge the limitations of this study. First, being a cross-sectional analysis, it does not allow for the establishment of a direct causal link between Lp(a) levels and thyroid hormone sensitivity. Additional studies are required to ascertain causality. Second, despite adjusting for multiple confounding factors, we cannot fully rule out the likelihood that serum Lp(a) levels might be impacted by other factors, such as dietary intake, smoking status, physical activity, socioeconomic status, as well as inflammatory or genetic markers, which we did not collect in this study. Third, our analysis, the participants were predominantly Chinese adults, and the data from a single tertiary hospital in China. This not only introduces genetic and ethnic homogeneity but may also introduce institutional or regional bias. Additionally, the sample was limited to patients with T2DM,which will limit the generalizability of the study findings.Furthermore, the size of the sample in this study is relatively small. Consequently, future research endeavors with larger sample sizes are necessitated.

## 5 Conclusion

This study demonstrated the association of reduced sensitivity to thyroid hormone with high Lp(a) level in euthyroid patients with T2DM. The results of this study emphasize the importance of monitoring thyroid hormone sensitivity and maintaining it at a good level to prevent the occurrence of dyslipidemia in euthyroid patients with T2DM, thereby reduce the overall burden of CVDs. Further research is needed to clarify the underlying mechanisms of this association and prospective cohort studies are needed to explore the causal relationship between reduced sensitivity to thyroid hormone and high Lp(a) level, to pave the way for potential therapeutic interventions targeting diabetes-related CVD risk.

## Data Availability

The raw data supporting the conclusions of this article will be made available by the authors, without undue reservation.

## Supplementary material

Supplementary Figure 1 Restricted spline curve of the FT3/FT4 ratio and TFQI odds ratio of high Lp(a) level of univariable logistic regression model. **(A)** The restricted spline curve of the FT3/FT4 ratio odds ratio of high Lp(a) level. **(B)** The restricted spline curve of the TFQI odds ratio of high Lp(a) level.

Supplementary Table 1 Univariable linear regression model for the association between Lp(a) levels and thyroid parameters in euthyroid patients with T2DM.

Supplementary Table 2 Univariable logistic regression model for the association between high Lp(a) level and thyroid parameters in euthyroid patients with T2DM.
