# Correction: Impaired sensitivity to thyroid hormones is associated with high lipoprotein(a) level in euthyroid patients with type 2 diabetes mellitus

**DOI:** 10.3389/fendo.2025.1654047

**Published:** 2025-07-11

**Authors:** Luojing Zhong, Ruiyu Lin, Baozhen Cao, Wenying Zhong, Mei Tu, Wen Wei

**Affiliations:** ^1^ Department of Endocrinology, Longyan First Affiliated Hospital of Fujian Medical University, Longyan, China; ^2^ Department of Physical Examination, Longyan First Affiliated Hospital of Fujian Medical University, Longyan, China; ^3^ The School of Clinical Medicine, Fujian Medical University, Fuzhou, China

**Keywords:** lipoprotein(a), sensitivity to thyroid hormones, type 2 diabetes mellitus, thyroid feedback quantile-based index, free triiodothyronine/free thyroxine ratio

In the published article, there was an error in **Figure 2D** as published. We found that the figure in **Figure 2C** and **Figure 2D** are duplicated, and the x-axis of **Figure 2D** should be TFQI. The corrected **Figure 2D** and its caption appear below.

**Figure 2:**
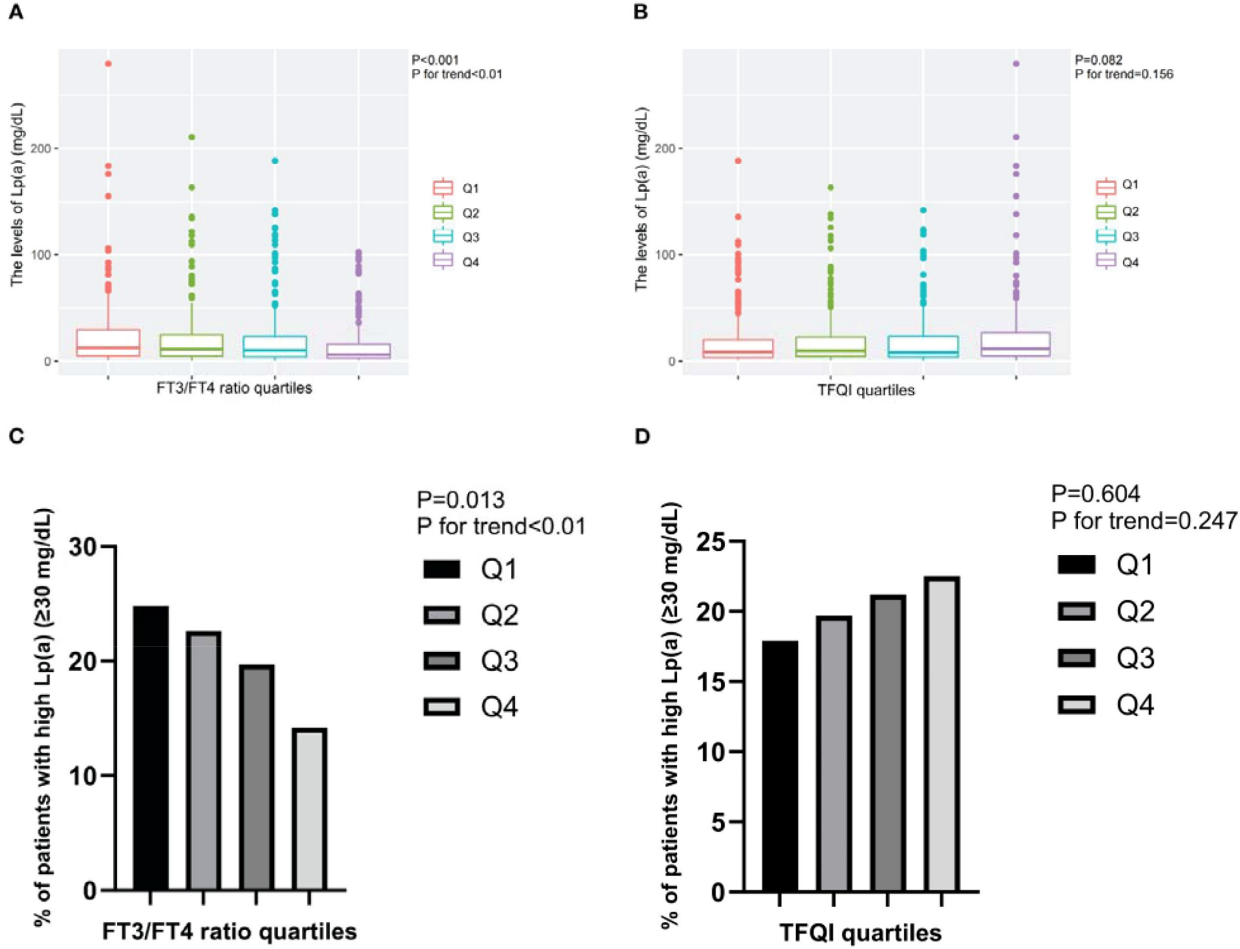
The levels of Lp(a) and the prevalence of T2DM patients with high Lp(a) level across the quartiles of thyroid parameters. **(A)** The levels of Lp(a) across the FT3/FT4 ratio quartiles. **(B)** The levels of Lp(a) across the TFQI quartiles. **(C)** The prevalence of T2DM patients with high Lp(a) level across the FT3/FT4 ratio quartiles. **(D)** The prevalence of T2DM patients with high Lp(a) level across the TFQI quartiles. Q1, the 1^st^ quartiles; Q2, the 2^nd^ quartiles; Q3, the 3^rd^ quartiles; Q4, the 4^th^ quartiles; Lp(a), lipoprotein(a); NS, P>0.05; FT3, Free Triiodothyronine; FT4, Free Thyroxine; TFQI, Thyroid feedback quantile-based index; T2DM, type 2 diabetes mellitus.

The original version of this article has been updated.

