# Supplementary material for: Impaired sensitivity to thyroid hormones is associated with high lipoprotein(a) level in euthyroid patients with type 2 diabetes mellitus
Source: Front Endocrinol (Lausanne). 2025 Jun 25;16:1591108. doi: 10.3389/fendo.2025.1591108 (PMC12238823; doi:10.3389/fendo.2025.1591108)
Supplement: Supplementary Figure 1 — Restricted spline curve of the FT3/FT4 ratio and TFQI odds ratio of high Lp(a) level of univariable logistic regression model. (A) The restricted spline curve of the FT3/FT4 ratio odds ratio of high Lp(a) level. (B) The restricted spline curve of the TFQI odds ratio of high Lp(a) level. [file DataSheet1.docx]

| **Variables** | **β (95% CI)** | ***P*-value** |
| --- | --- | --- |
| Age | 0.05(-0.09,0.18) | 0.498 |
| Sex | -3.65(-7.05,-0.26) | 0.035 |
| BMI | -0.85(-1.35,-0.36) | 0.001 |
| Hypertension | -0.87(-4.17,2.43) | 0.604 |
| SBP | 0.05(-0.04,0.14) | 0.280 |
| DBP | -0.10(-0.24,0.05) | 0.188 |
| Duration of diabetes | 0.14(-0.09,0.38) | 0.235 |
| DR | 4.18(0.36,8.00) | 0.032 |
| DN | 3.64(-1.25,8.52) | 0.144 |
| DPN | -0.01(-3.44,3.42) | 0.997 |
| CVD | -1.49(-6.70,3.72) | 0.574 |
| FBG | -0.20(-0.71,0.32) | 0.452 |
| HbA1C | 0.58(-0.15,1.30) | 0.119 |
| ALT | -0.11(-0.18,-0.03) | 0.005 |
| AST | -0.14(-0.23,-0.05) | 0.003 |
| Creatinine | 0.01(-0.03,0.05) | 0.627 |
| eGFR | -0.06(-0.13,0.01) | 0.091 |
| TC | 1.58(0.45,2.71) | 0.006 |
| TG | 1.45(0.77,2.14) | 0.000 |
| LDL-C | 3.21(1.53,4.88) | 0.000 |
| HDL-C | -11.90(-17.60,-6.19) | 0.000 |
| FT3 | -1.91(-4.50,0.67) | 0.147 |
| FT4 | 1.21(0.26,2.17) | 0.013 |
| TSH | -0.38(-1.94,1.18) | 0.634 |
| FT3/FT4 | -31.12(-51.70,-10.55) | 0.003 |
| TT4RI | 0.03(-0.11,0.17) | 0.689 |
| TSHI | 1.87(-1.25,4.98) | 0.240 |
| TFQI | 4.26(0.10,8.24) | 0.049 |

**Table S1 Univariable linear regression model for the association between Lp(a) levels and thyroid parameters in euthyroid patients with T2DM**

**Abbreviations**:Lp(a), lipoprotein(a); BMI, body mass index;SBP,systolic blood pressure; DBP,di-astolic blood pressure;DR, diabetic retinopathy; DN, diabetic nephropathy; DPN, diabetic periphe-ral neuropathy; CVD, cardiovascular disease;FBG, fasting blood glucose;HbA1c, glycosylated he-moglobin; AST, aspartate aminotransferase; ALT, alanine aminotransferase; eGFR,estimated glo-

merular filtration rate;TG, triglyceride;TC, total cholesterol; LDL-C, low-density lipoprotein cho-

lesterol; HDL-C, high-density lipoprotein cholesterol;FT3, Free Triiodothyronine; FT4, Free Thyr-oxine ; TSH, Thyroid-stimulating hormone; TSHI,Thyroid-stimulating hormone index; TT4RI, T-hyrotrophic T4 resistance index; TFQI, Thyroid feedback quantile-based index; T2DM, type 2 di- abetes mellitus.

| **Variables** | **OR (95% CI)** | ***P*-value** |
| --- | --- | --- |
| Age | 1.00(0.99,1.01) | 0.904 |
| Sex | 1.04(0.77,1.41) | 0.812 |
| BMI | 0.95(0.91,1.00) | 0.046 |
| Hypertension | 0.99(0.74,1.33) | 0.946 |
| SBP | 1.00(0.99,1.01) | 0.986 |
| DBP | 0.99(0.98,1.01) | 0.280 |
| Duration of diabetes | 1.01(0.99,1.03) | 0.598 |
| DR | 1.22(0.87,1.71) | 0.243 |
| DN | 1.32(0.86,1.98) | 0.189 |
| DPN | 1.02(0.75,1.38) | 0.896 |
| CVD | 1.06(0.66,1.65) | 0.813 |
| FBG | 0.98(0.93,1.02) | 0.357 |
| HbA1C | 1.05(0.99,1.12) | 0.114 |
| ALT | 0.99(0.99,1.00) | 0.144 |
| AST | 0.99(0.98,1.00) | 0.049 |
| Creatinine | 1.00(1.00,1.01) | 0.178 |
| eGFR | 0.99(0.99,1.00) | 0.034 |
| TC | 1.11(1.00,1.21) | 0.045 |
| TG | 1.10(1.02,1.21) | 0.023 |
| LDL-C | 1.22(1.05,1.41) | 0.008 |
| HDL-C | 0.58(0.35,0.96) | 0.031 |
| Statin | 1.43(0.91,2.20) | 0.114 |
| Fibrate | 2.01(0.28,10.36) | 0.423 |
| Aspirin/Clopidogrel | 1.51(0.91,2.43) | 0.096 |
| FT3 | 0.87(0.69,1.10) | 0.240 |
| FT4 | 1.13(1.04,1.23) | 0.004 |
| TSH | 0.92(0.79,1.06) | 0.244 |
| FT3/FT4 | 0.05(0.01,0.35) | 0.003 |
| TT4RI | 1.00(0.99,1.01) | 0.768 |
| TSHI | 1.05(0.79,1.39) | 0.734 |
| TFQI | 1.51(1.01,2.30) | 0.047 |

**Table S2 Univariable logistic regression model for the association between high Lp(a) level and thyroid parameters in euthyroid patients with T2DM**

**Abbreviations**:Lp(a), lipoprotein(a); BMI, body mass index;SBP,systolic blood pressure; DBP,di-astolic blood pressure;DR, diabetic retinopathy; DN, diabetic nephropathy; DPN, diabetic periphe-ral neuropathy; CVD, cardiovascular disease;FBG, fasting blood glucose;HbA1c, glycosylated he-moglobin; AST, aspartate aminotransferase; ALT, alanine aminotransferase; eGFR,estimated glo-

merular filtration rate;TG, triglyceride;TC, total cholesterol; LDL-C, low-density lipoprotein cho-

lesterol; HDL-C, high-density lipoprotein cholesterol;FT3, Free Triiodothyronine; FT4, Free Thyr-oxine ; TSH, Thyroid-stimulating hormone; TSHI,Thyroid-stimulating hormone index; TT4RI, T-hyrotrophic T4 resistance index; TFQI, Thyroid feedback quantile-based index; T2DM, type 2 di- abetes mellitus.

| 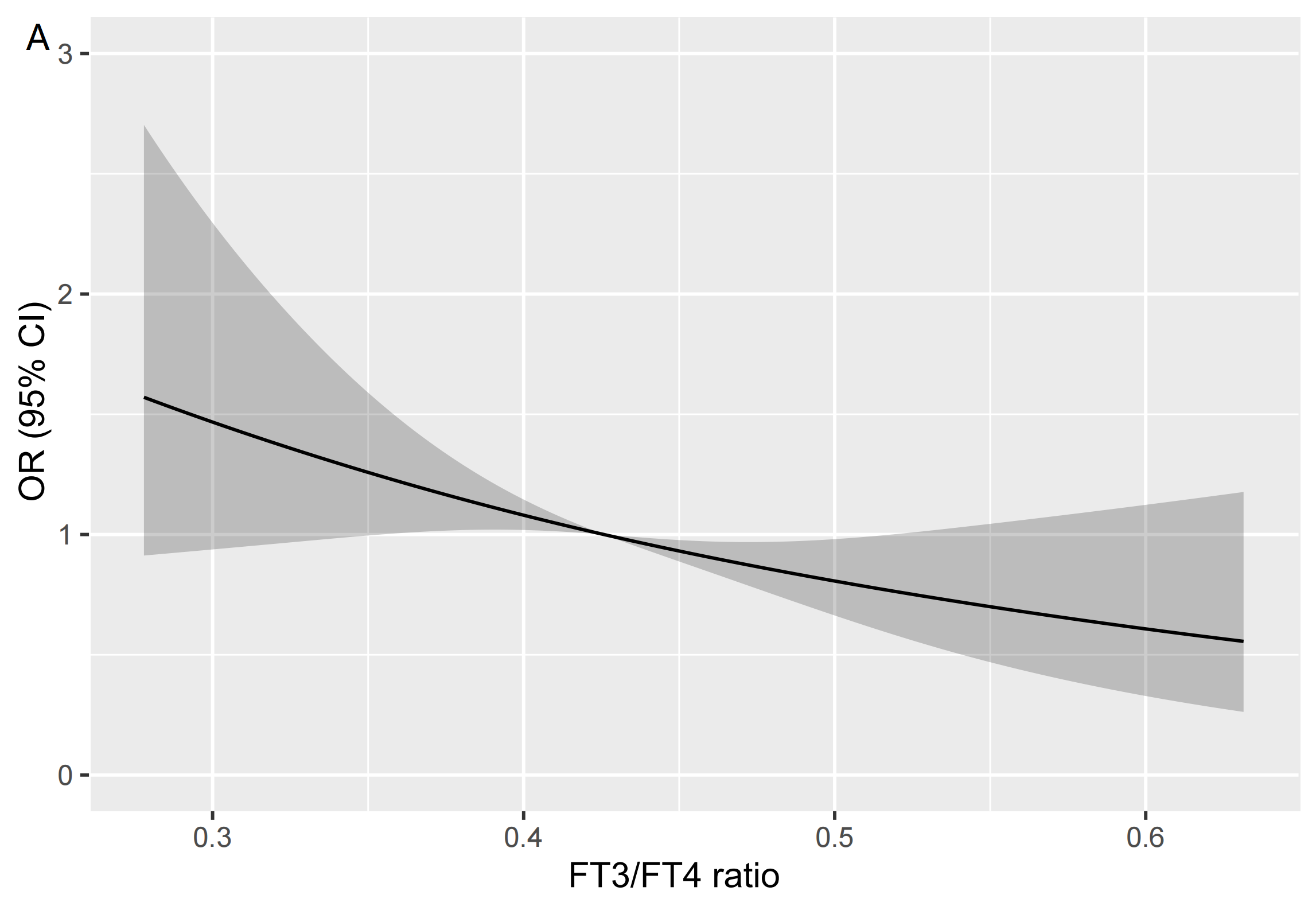 | 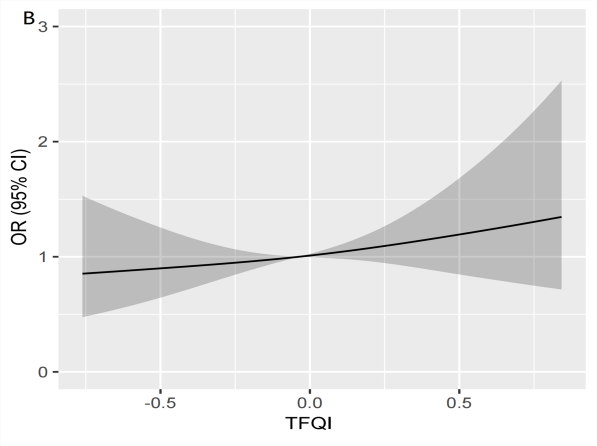 |
| --- | --- |

**Figure S1** Restricted spline curve of the FT3/FT4 ratio and TFQI odds ratio of high Lp(a) level of univariable logistic regression model. **(A)**The restricted spline curve of the FT3/FT4 ratio odds ratio of high Lp(a) level.

**(B)**The restricted spline curve of the TFQI odds ratio of high Lp(a) level.

**Abbreviations**:Lp(a), lipoprotein(a); FT3, Free Triiodothyronine; FT4, Free Thyroxine; TFQI, Thyroid feed-

back quantile-based index.
